# Beyond the Usual Suspects: Primary Premaxilla Sarcoidosis

**DOI:** 10.22038/IJORL.2023.73996.3493

**Published:** 2024-01

**Authors:** Pankhuri Mittal, Brijnandan Gupta, Subodh Kumar, Aakanksha Rawat, Anshi Singh

**Affiliations:** 1 *Department of ENT, All India Institute of Medical Sciences (AIIMS) Gorakhpur, India. *; 2 *Department of Pathology, AIIMS Gorakhpur, India. *; 3 *Department of Pulmonary Medicine, AIIMS Gorakhpur, India.*

**Keywords:** Sarcoidosis, Head and Neck Sarcoidosis, Extra-pulmonary sarcoidosis

## Abstract

**Introduction:**

Sarcoidosis is an idiopathic systemic granulomatous disorder that can affect multiple organs, including rare extrapulmonary sites like the premaxilla. This case report presents a rare occurrence of premaxillary sarcoidosis, a condition scarcely reported in medical literature.

**Case Report:**

The patient, a 62-year-old male, presented with a progressively enlarging painless swelling on the right cheek over a three-year period. Despite multiple Fine Needle Aspiration Cytology (FNAC) examinations yielding no conclusive diagnosis, a contrast-enhanced computed tomographic (CT) scan revealed an ill-defined lesion in the premaxillary soft tissue. Biopsy and subsequent excision procedures confirmed the presence of non-caseating granulomas with asteroid bodies, indicative of sarcoidosis. With no systemic involvement and complete excision of the disease, further treatment was not necessary.

**Conclusion:**

This case highlights the challenges in diagnosing premaxillary (Extrapulmonary Sarcoidosis) sarcoidosis due to its rarity and resemblance to other dental and maxillofacial conditions and granulomatous lesions. Accurate diagnosis requires a high index of suspicion, multidisciplinary approach, involving clinical assessment, histopathological analysis, and imaging modalities. By deepening our understanding of these uncommon presentations, this report aims to enhance clinical awareness and contribute to improved patient outcomes.

## Introduction

Sarcoidosis, an idiopathic systemic granulomatous disorder, presents with diverse clinical manifestations and an unpredictable course (1). While sarcoidosis can involve multiple organs, extrapulmonary sites are often not spared, leading to a wide range of symptoms and complications (2). Within this context, rare cases emerge that challenge our understanding of sarcoidosis. In this particular instance, we explore an exceedingly rare occurrence involving the premaxilla, an entity scarcely reported in medical literature posing a dilemma of diagnosis.

Sarcoidosis is characterised by the formation of non-caseating granulomas, primarily affecting the lungs and intrathoracic lymph nodes (3). However, it can involve virtually any organ system, such as the skin, eyes, liver, heart, and central nervous system (4). While sarcoidosis involving the premaxillary space is exceptionally rare, it is crucial to recognize that other granulomatous conditions may also affect this anatomical site.

The premaxilla plays a pivotal role in facial aesthetics and dental occlusion. Granulomatous conditions involving the premaxilla pose unique challenges for diagnosis and management, as the inflammation can disrupt the delicate balance of oral and maxillofacial structures. As a result, patients may present with a spectrum of clinical features, ranging from radiological abnormalities to significant facial deformities, pain, dental abnormalities, and functional impairment. Besides sarcoidosis, other granulomatous conditions may involve the premaxilla. These conditions include tuberculosis, Wegener's granulomatosis, midline granuloma, foreign body granuloma, and granulomatosis with polyangiitis (formerly known as Wegener's granulomatosis). Each condition exhibits distinct clinical and histopathological features that require careful evaluation to establish an accurate diagnosis (5).

Given the rarity of premaxillary involvement in granulomatous conditions, the etiopathogenesis and optimal treatment strategies remain poorly defined. Accurate diagnosis often necessitates a multidisciplinary approach, involving detailed clinical assessment, histopathological analysis, and integration of various imaging modalities to guide appropriate management decisions (6).

This case report aims to shed light on the intricate interplay between granulomatous conditions and the premaxilla. By deepening our understanding of these uncommon presentations, we hope to enhance clinical awareness and contribute to improved patient outcomes.

## Case Report

A 62-year-old male patient presented to our outpatient department with a progressively enlarging painless swelling on the right cheek over a period of three years. The patient denied any previous history of sinus or nasal-related issues. Upon examination, we noted a well-defined, firm swelling in the right premaxillary region, accompanied by a slight bulge in the upper sublabial area. Nasal endoscopy findings were unremarkable and there was no cervical lymphadenopathy. Systemic examination was normal. It is noteworthy that the patient had undergone multiple Fine Needle Aspiration Cytology (FNAC) examinations at different hospitals, all of which failed to yield any conclusive diagnosis.

**Fig 1:**
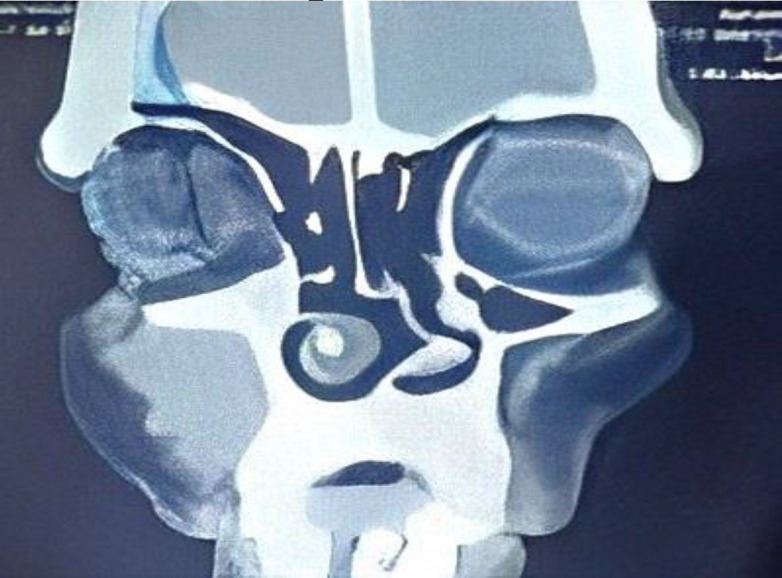
3.3 cm x 3 cm, lesion with heterogeneous enhancement with osteo-destruction

A contrast-enhanced computed tomographic (CT) scan was performed to evaluate the nose and paranasal sinuses, revealing an ill-defined lesion in the premaxillary soft tissue. The lesion, measuring approximately 3.3 cm x 3 cm, exhibited heterogeneous enhancement and displayed features consistent with osteo-destruction. Erosion of the anterior wall of the maxillary sinus underlying the lesion was observed, without any evidence of cortical breach. Importantly, the maxillary sinus itself was unaffected, and there were no indications of extension into the orbital or intracranial regions (Figure 1). The patient had a history of asthma and had been using inhalers for the past five years. All routine blood parameters were within normal ranges.

Considering the duration of the lesion (three years) and the CT findings, several differential diagnoses were considered, including tuberculosis, fungal granuloma, giant reparative granuloma, Wegener's granulomatosis, sarcoidosis, and histiocytoma.

To confirm the diagnosis histologically, a biopsy was performed using a sublabial approach. The histopathological examination revealed the presence of multiple epithelioid cell granulomas composed of epithelioid cells, lymphocytes, and Langhans giant cells. Ziehl-Neelsen staining for Acid-fast bacilli (AFB) was negative.

Based on the presence of non-caseating granulomas and negative AFB staining, the possibility of infective conditions (such as tuberculosis or fungal infection) was considered less likely. However, due to the absence of a definitive diagnosis, an excision biopsy was scheduled to obtain a more precise understanding of the condition to guide further management if needed.

**Fig 2:**
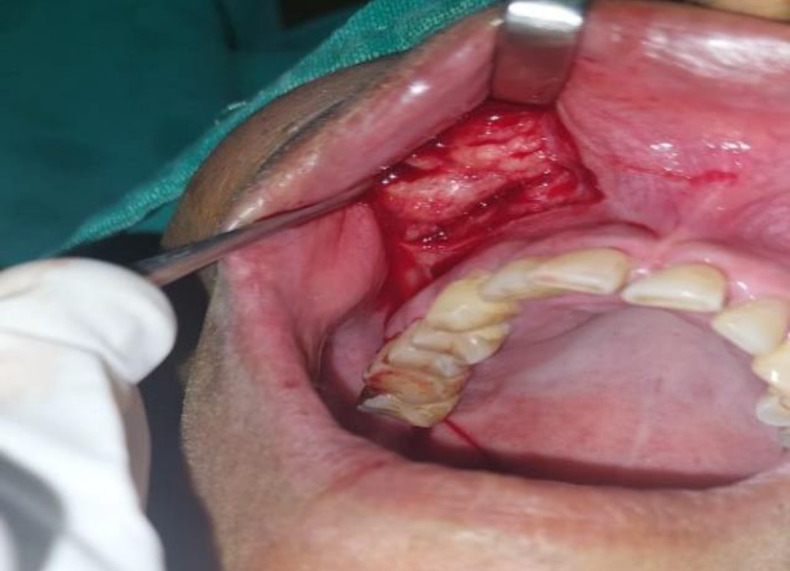
Clinical picture showing whitish firm lesion as seen during excision via sub-labial approach

The patient underwent an excision procedure using a sublabial approach. During the surgery, a whitish firm mass measuring 4 cm x 3 cm was identified in the premaxilla and completely removed (Figure 2).

**Fig 3:**
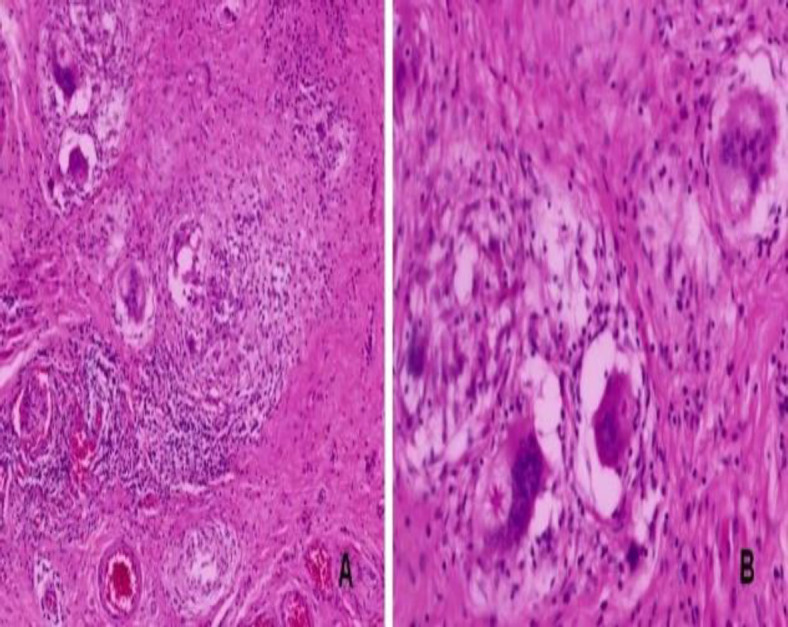
A. Excision specimen showed epithelioid cell granuloma and multinucleated giant cells having Asteroid bodies (Hematoxylin and Eosin x100) B. High power image showing multinucleated giant cells having eosinophilic, stellate shaped Asteroid bodies (Hematoxylin and Eosin x400)

Additionally, the eroded underlying anterior wall of the maxilla was excised. Importantly, the mucosa of the underlying maxillary sinus was found to be uninvolved. The excised specimen was sent for histopathological evaluation. To our surprise, the histology this time revealed the presence of a non-caseating granuloma characterised by the presence of epithelioid cells, giant cells, and some giant cells exhibiting Asteroid bodies suggestive of sarcoidosis! (Figure 3).

Although the patient's serum ACE level was within the normal range, the serum total calcium level was high normal. The 24-hour urinary calcium level was found to be within the normal range. In order to investigate the possibility of lung involvement, a pulmonary medicine consultation t was sought. A CT scan of the thorax was conducted, and the findings revealed no abnormality. 

A bronchoscopic evaluation also revealed normal tracheobronchial tree and normal bronchial mucosa appearance. A transbronchial lung biopsy nonetheless was taken from a suspicious area which was normal. 

The patient was ultimately diagnosed with primary premaxillary sarcoidosis. Considering the absence of systemic involvement and the complete excision of the disease, no further treatment was deemed necessary. The patient has been closely monitored through regular follow-ups with our team and pulmonary medicine specialists for the past six months. Encouragingly, the patient's condition has shown significant improvement, and he is currently in excellent health with no notable complications.

## Discussion

Sarcoidosis patients can exhibit a wide range of symptoms depending on the organs affected (7). The most commonly involved areas include the lungs, eyes, and skin. Pulmonary involvement, observed in approximately 90% of cases, is characterised by manifestations such as hilar lymphadenopathy and pulmonary infiltration (8). Head and neck involvement arises in 10%-15% of sarcoidosis cases (9). 

Oral manifestations are uncommon, with fewer than 80 documented cases (10). The salivary glands, notably the parotid glands, are frequently affected, leading to a 6% incidence of enlarged parotids due to granulomatous infiltration, presenting as xerostomia (11,12). Oral signs can manifest as singular or multiple lesions on the buccal mucosa, tongue, or lips, appearing as brown/red papules, nodules, or fine granular macules (13). Bone involvement occurs in 5%-15% of cases, commonly affecting the small tubular bones of the hands and feet, while maxillofacial and skull base involvement is exceedingly rare (13,14). A comprehensive study in Spain involving 425 patients reported only one case involving the skull (15,16).

Diagnosing head and neck sarcoidosis is challenging due to the lack of definitive features. Besides histological findings, specific clinical symptoms and ruling out other similar inflammatory conditions are crucial for confirmation. Pulmonary involvement is an exception, where non-caseating granulomatous tissue in one organ is enough for diagnosis. If other biopsies are inconclusive, a lung parenchyma or lymph node sample is taken. Histologically, granulomatous inflammation without caseating necrosis is observed, often with specific inclusion bodies. Considering other potential causes of granulomatous lesions is vital. While no specific markers exist, tests like ACE levels can offer diagnostic clues. However, elevated ACE levels lack specificity and can be high in other diseases. Interpretation alongside other tests is necessary. Elevated ACE levels may suggest active disease and aid in monitoring disease progression (17-19).

## Conclusion

The diagnosis of premaxillary sarcoidosis poses a challenge due to its rarity and resemblance to other dental and maxillofacial conditions. The nonspecific clinical presentation and overlapping radiographic features often lead to misdiagnosis and delayed treatment. It is crucial to consider sarcoidosis in the differential diagnosis when encountering premaxillary swelling, bone involvement, or refractory dental infections. Given the rarity of premaxillary involvement in granulomatous conditions, the exact cause and optimal treatment strategies remain poorly defined. Accurate diagnosis typically requires a multidisciplinary approach, involving thorough clinical assessment, histopathological analysis, and integration of various imaging modalities to guide appropriate management decisions.
